# The Respiratory Microbiome in Cystic Fibrosis: Compartment Patterns and Clinical Relationships in Early Stage Disease

**DOI:** 10.3389/fmicb.2020.01463

**Published:** 2020-06-30

**Authors:** Marian Garcia-Nuñez, Miguel Garcia-Gonzalez, Xavier Pomares, Concepción Montón, Laura Millares, Sara Quero, Elena Prina, Oscar Asensio, Montserrat Bosque, Silvia Capilla, Oscar Cuevas, Eduard Monsó

**Affiliations:** ^1^Department of Respiratory Medicine, Institut d’Investigació i Innovació Parc Taulí (I3PT), Hospital Universitari Parc Taulí, Sabadell, Spain; ^2^Centro de Investigación en Red en Enfermedades Respiratorias (CIBERES), Instituto de Salud Carlos III, Madrid, Spain; ^3^Cystic Fibrosis Unit, Hospital Universitari Parc Taulí, Sabadell, Spain; ^4^Department of Pediatrics, Institut d’Investigació i Innovació Parc Taulí (I3PT), Hospital Universitari Parc Taulí, Sabadell, Spain; ^5^Universitat Autonoma de Barcelona, Barcelona, Spain; ^6^Infectious and Respiratory Disease Research Group, Fundació Institut d’Investigació Germans Trias i Pujol, Badalona, Spain; ^7^Department of Microbiology, Hospital Universitari Parc Taulí, Sabadell, Spain

**Keywords:** cystic fibrosis, microbiome, exacerbation, lung function, *Staphylococcus aureus*

## Abstract

We compared the bacterial microbiomes lodged in the bronchial tree, oropharynx and nose of patients with early stage cystic fibrosis (CF) not using chronic antibiotics, determining their relationships with lung function and exacerbation frequency. CF patients were enrolled in a cohort study during stability and were checked regularly over the following 9 months. Upper respiratory samples (sputum [S], oropharyngeal swab [OP] and nasal washing [N]) were collected at the first visit and every 3 months. 16S rRNA gene amplification and sequencing was performed and analyzed with QIIME. Seventeen CF patients were enrolled (16.6 SD 9.6 years). Alpha-diversity of bacterial communities between samples was significantly higher in S than in OP (Shannon index median 4.6 [IQR: 4.1–4.9] vs. 3.7 [IQR: 3-1-4.1], *p* = 0.003/Chao 1 richness estimator median 97.75 [IQR: 85.1–110.9] vs. 43.9 [IQR: 31.7–59.9], *p* = 0.003) and beta-diversity analysis also showed significant differences in the microbial composition of both respiratory compartments (Adonis test of Bray Curtis dissimilarity matrix, *p* = 0.001). Dominant taxa were found at baseline in five patients (29.4%), who showed lower forced expiratory volume in the first second (FEV1%, mean 74.8 [SD 19] vs. 97.2 [SD 17.8], *p* = 0.035, Student *t* test). The *Staphylococcus* genus had low RAs in most samples (median 0.26% [IQR 0.01–0.69%]), but patients with RA > 0.26% of Staphylococcus in bronchial secretions suffered more exacerbations during follow-up (median 2 [IQR 1–2.25] vs. 0 [0–1], *p* = 0.026. Mann–Whitney U test), due to *S. aureus* in more than a half of the cases, microorganism that often persists as bronchial colonized in these patients (9/10 [90%] vs. 2/7 [28.6%], *p* = 0.034, Fisher’s exact test). In conclusion, the bronchial microbiome had significantly higher diversity than the microbial flora lodged in the oropharynx in early stage CF. Although the RA of the *Staphylococcus* genus was low in bronchial secretions and did not reach a dominance pattern, slight overrepresentations of this genus was associated with higher exacerbation frequencies in these patients.

## Introduction

Cystic fibrosis (CF) is a chronic respiratory disease characterized by defective mucociliary clearance and recurrent infections by various potentially pathogenic microorganisms (PPMs) such as *Pseudomonas aeruginosa*, *Staphylococcus aureus*, *Haemophilus influenzae*, *Stenotrophomonas maltophila*, *Achromobacter xylosoxidans* and the *Burkholderia cepacia* complex, some of which evolve into chronic colonization (Guss et al., 2011; Sibley et al., 2011). Culture-independent approaches have recently been introduced in the analysis of the respiratory microbiology in CF and have demonstrated that sputum (S) and oropharyngeal (OP) cultures only identify a fraction of the microbial flora lodged in the bronchial tree and the oropharynx, missing a wide range of bacteria, which include anaerobic genera as *Prevotella*, *Veillonella*, *Fusobacterium*, and *Streptococcus*, that required specific cultures (Guss et al., 2011; Sibley et al., 2011; Fodor et al., 2012; Carmody et al., 2018). The diversity of this respiratory microbiome has a clinical meaning in CF, because its decline, mainly due to the progressive loss of anaerobic flora and its partial substitution by PPMs that increase their relative abundance, has been related to the lung function impairment commonly found in the disease (Delhaes et al., 2012; Fodor et al., 2012; Flight et al., 2015; Carmody et al., 2018).

OP samples are easily obtained in CF patients and have been often considered representative of bronchial secretions, and recent series have shown a close similarity of the microbiomes in bronchi and oropharynx (Fodor et al., 2012; Boutin et al., 2015). These studies have enrolled a restricted number of cases and included a high proportion of patients at intermediate or advanced stages of the disease (FEV1% < 70%) and/or chronic *P. aeruginosa* colonization. The relationships between the microbial composition of these upper airway sites and their clinical meaningfulness in early stages of the disease are mostly unknown.

The objectives of this cohort study were to compare the bacterial microbiomes at different sites of the upper respiratory airway of early stage CF patients (FEV1% ≥ 70), to determine its regional and temporal variability, and their relationships with clinical patterns of the disease, focusing on lung function and exacerbation frequency. Samples from sputum (S), oropharyngeal swabs (OP) and nasal lavages (N) were examined and compared both cross-sectionally and longitudinally to attain the aims of the study.

## Materials and Methods

### Design and Patients

Between March 2013 and November 2014 CF all patients who regularly attended a reference university hospital and were able to produce spontaneous sputum were enrolled in the cohort when clinically stable, and regularly checked every 3 months over the following 9 months outside exacerbations. Clinical data, spirometry results and respiratory samples (S, OP and N) for microbiome analysis were collected at baseline, and followed by the collection of S at scheduled stability visits till the 9th month of follow-up. A second collection of OP was also obtained 6 months after the first visit. Absence of acute symptoms, functional impairment or antibiotic treatments in the previous month were the stability criteria required before every sampling. Patients treated with chronic inhaled or systemic antibiotics at the moment of enrolment were not included in the study. None of the patients used modulators during the study period. Acute episodes of increased sputum production and/or dyspnea during follow-up were identified as exacerbations when they required antibiotics, and additional S samples were obtained for usual care culture in these episodes. Participants with two or more exacerbations recorded during the study were considered frequent exacerbators.

### Sociodemographic and Clinical Measurements

Sociodemographic and clinical data, diagnostic tests and treatments were recorded at enrollment. All patients performed a forced spirometry with reversibility testing at this first visit, in the morning, with a dry rolling-seal spirometer (Sibelmed, Sibelgroup, Barcelona, Spain), in accordance with standard techniques (American Thoracic Society, 1995). Postbronchodilator forced vital capacity (FVC) and forced expiratory volume in the first second (FEV1) were measured and compared to age and height-adjusted reference values. Patients with FEV1% ≥ 70 were considered at early stage, while values ≥ 40–70 and <40 were identified as suffering from intermediate and advanced disease, respectively (Caverly et al., 2019).

### Sample Acquisition and Processing

Spontaneous S samples were collected in a sterile container and a part of it was processed as usual care and cultured on agar plates (MacConkey agar, chocolate agar Polyvitex, Columbia agar + 5% sheep blood, Columbia CAN agar + 5% sheep blood and Saboraud Chloramphenicol agar). MacConkey cultures were incubated at 36°C, Saboraud Chloramphenicol agar at 30°C and all the other agar plates at 36°C in CO_2_ enriched conditions. Recovered bacteria were identified through mass spectrometry using MALDI-TOF Biotyper version 3.0 (Bruker Daltonics, Billerica, MA, United States), reporting only predominant PPMs. Patients with at least one positive S culture for a PPM during the study, and who had three or more positive results for that PPM during this period and/or the previous year were considered chronically colonized (Döring et al., 2000; Lee et al., 2003; Junge et al., 2016). The remnant of the S sample after the culture was stored for microbiome analyses. OP samples were obtained with a sterile cotton swab (Copan Diagnostics Inc., Murrieta, CA, United States) from the posterior oropharynx, and N samples through a sterile Foley catheter after flushing 10 mL of pyrogenic free saline 0.9% (Fresenius Kabi, Denmark) and recovery through aspiration. Sterile control samples were also collected for each type of sample: saline on unused S containers and after instillation and aspiration through a nasal catheter, and unused swabs. These samples were handled aseptically and processed in parallel with the clinical specimens. All samples with their controls were stored at −80°C until use.

### DNA Extraction

Samples were manipulated in biosafety level 2 hoods with laminar flow. All samples were thawed on ice prior to DNA extraction. S samples were first incubated with four volumes of Sputasol (Oxoid, Hampshire, United Kingdom), mixed with four volumes of phosphate buffer solution and centrifuged at 16000 *g* for 10 min. The pellet obtained was mixed with 0.6 mL of a lysis buffer containing lysozime (final concentration 83 μg/mL; Sigma-Aldrich Corp., St. Louis, MO, United States), lysostaphin (final concentration 20 U/mL; Sigma-Aldrich) and mutanolysin (final concentration 250 U/mL; Sigma-Aldrich). OP samples were directly suspended on the same volume of the lysis buffer. The same procedure was followed with the pellet obtained from N samples after centrifugation. All samples were incubated during 2 h at 37°C (Yuan et al., 2012).

Samples were transferred to a DNA dry bead tube (MO BIO Laboratories, Carlsbad, CA, United States), and shaken at 5000 rpm for 30 s in a Precellys Minilys homogenizer (Bertin Technologies, Rockville, Washington, DC, United States). After digesting with Proteinase K for 1 h at 60°C, samples were again shaken in the homogenizer and DNA was purified according to the manufacturer’s instructions. Genomic DNAs were quantified using the Qubit Fluorometer (Life Technologies. Carlsbad, CA, United States).

### PCR Amplification and Sequencing of 16S rRNA Gene

The 16S rDNA V3-V4 region was amplified following the 16S Metagenomic Sequencing Library Preparation Illumina protocol (Part # 15044223 Rev. A; Illumina, CA, United States). Illumina adapter overhang nucleotide sequences were added to gene-specific sequences, selecting primers following Klindworth and cols (Klindworth et al., 2013). Using the standard International Union of Pure and Applied Chemistry nucleotide nomenclature, the full-length primer sequences used to follow the protocol targeting this region were: 16S Forward primer = 5′- tcgtcggcagcgtcagatgtgtataagagacagcctacgggnggcwgcag-3′ and reverse primer = 5′-gtctcgtgggctcggagatgtgtataagagacaggactachvg ggtatctaatcc-3′. After 16S amplification, the multiplexing step was performed using Nextera XT Index Kit (FC-131-1096, Illumina). Extraction controls were amplified in parallel with the samples. Libraries were sequenced using a 2 × 300 bp paired-end run (MiSeq Reagent kit v3 MS-102-3001, Illumina), on a MiSeq Sequencer in accordance with manufacturer’s instructions (Illumina). Quality assessment was performed by the use of the PRINSEQ-lite program (Schmieder and Edwards, 2011), on the following parameters: min_length: 50; trim_qual_right: 20; trim_qual_type: mean; trim_qual_window: 20. R1 and R2 from Illumina sequencing were joined using the fastq-join program, part of the ea-tools suite (Aronesty, 2011).

### Sequence Analysis

The Quantitative Insights Into Microbial Ecology (QIIME) pipeline 1.9.0 (Caporaso et al., 2010), was used for sequence processing to obtain taxonomic information using Greengenes 13_8 sequence database as reference and RDP classifier 2.2. The open reference operational taxonomic unit (OTU) picking method was used with UCLUST and PyNAST version 1.2.2 as alignment method. Chimeric sequences were detected in QIIME with ChimeraSlayer and were removed from the OTU table and the phylogenetic tree to perform downstream analysis.

### Identification of Procedural Contaminants

To assess the influence of the procedure and reagent contamination in our samples, we sequenced 6 extraction controls and one PCR negative control. We obtained a mean of 85 (SD 10) sequences in these controls, which were also processed in QIIME. Eighty-two genera were identified in these negative controls, 75 of which were also present in the clinical samples. The number of reads per sample was significantly lower in control samples compared to N, OP, S samples (Kruskal–Wallis test, *p* < 0.001).

Identified genera were removed from the samples if their relative abundances (RAs) were higher in controls (Mann–Whitney U test) or showed a significantly higher prevalence in them (Fisher’s exact test) (Bittinger et al., 2014). In low bacterial biomass samples (N and OP), bacterial genera detected with RAs inversely correlated with the total bacterial DNA quantified in the amplicons (Spearman’s rho test) were also considered as contaminants and discarded (Jervis-Bardy et al., 2015). Downstream analyses to determine alpha and beta-diversity were performed after removing contaminant OTUs from the final OTU tables.

### Data Analysis

Statistical analyses were performed using SPSS statistical software version 19 (IBM SPSS Statistics, Armonk, NY, United States) and R packages. Descriptive analyses obtained from categorical variables are expressed as absolute and relative frequencies and results for continuous variables as means and standard deviations (SD) when the distribution was normal, or as medians and interquartile range (IQR) otherwise.

Alpha diversity in bronchi, oropharynx and nose was calculated through Shannon index (Shannon, 1997) and Chao 1 richness estimator (Chao, 1984) after subsampling with QIIME to avoid sequencing effort bias. Beta diversity and differences between the three compartments was assessed through Principal Coordinates Analysis (PCoA) with Bray-Curtis dissimilarity index (Bray and Curtis, 1957) and Adonis testing. Dissimilarity distances (θ_*YC*_ distance) among paired samples of S and OP were also assessed (Yue and Clayton, 2005).

Samples were normalized to the percentage of total reads for the comparisons of their RAs. The RA of each taxon was calculated by dividing its number of sequences by the total number of sequences in the sample. A dominant genus in a sample was defined as the most abundant genus with at least twice the abundance of the second most abundant genus (Coburn et al., 2015), and the identification of dominance either at the first or its immediately successive sample was considered as baseline dominance pattern.

First, genetic characteristics, baseline lung function, previous and subsequent exacerbations and culture-based microbiology were assessed. Second, alpha- and beta-diversity of the bacterial flora in bronchi, oropharynx and nose were examined and compared in both baseline and longitudinal samples. Finally, relationships between the RAs of the identified genera in S and clinical patterns were assessed, considering the median of RAs at the baseline and its successive value as the reference (cut-off) RA for these analyses.

For differential abundance analyses between groups, taxa with median RAs above 0.1% in at least one type of sample were included. Student *t* test, chi-square and Fisher’s exact test, together with non-parametric Mann-Whitney U, Wilcoxon signed rank, Friedman and Kruskal–Wallis tests were used for testing, as required. Correction for multiple hypothesis testing was done by computing false discovery rates (FDR). Tests producing a *P* value equal to or less than 0.05 were considered as significant.

## Results

### Patient Characteristics

Respiratory samples were collected prospectively from 17 patients with CF in stable phase (6 adults and 11 children). Sixty-eight S samples were collected, as well as 33 OP and 17 N samples obtained on the same day as an S sample. Patients had a median age of 13 (IQR: 11–20) years and most presented delta F508 gene mutations (15 patients, 88.2%, 14/1 hetero/homozygous). Mean sweat test chloride at diagnosis was 95.2 (SD 20) mEq/L. Twelve patients had lung volumes within normal ranges at enrollment (70.6%). One patient had used nebulized amikacin for more than 4 weeks the year before inclusion, but not during the month previous to enrollment (Table 1).

**TABLE 1:** Patient characteristics at baseline.

**N**	**17**
Age (years), median (IQR)	13 (11–20)
Gender (male), *n* (%)	9 (52.9)
Sweat test (Cl- mEq/L), mean (SD)	95.2 (20)
CFTR genotype, *n* (%)	
ΔF508-homozygous	3 (17.6)
ΔF508-heterozygous	12 (70.6)
Other mutation	2 (11.7)
FEV_1_% predicted, mean (SD)	90.6 (20.5)
FVC% predicted, mean (SD)	92.9 (19.1)
FEV1/FVC%, mean (SD)	91.9 (12.3)
Use of nebulized antibiotic during the previous year (>1 month), *n* (%)	1 (5.9)
Exacerbations in the previous year, median (IQR)	1 (0–1)

*IQR, interquartile range; SD, standard deviation; CFTR, Cystic fibrosis transmembrane conductance regulator.*

Bacterial cultures of S were positive of *S. aureus* in six patients at baseline (35.3%), and positive results for this PPM were found in follow-up stability samples from 5 additional patients who had previous negative S cultures (29.4%). These 11 patients showed recurrently positive cultures and attained the criteria for chronic colonization by *S. aureus* (64.7%), but repeatedly positive cultures were not found for other PPMs.

Positive cultures for *S. aureus* at enrolment were not significantly associated to FEV1 impairment (FEV1% mean 79.8 [SD 25.1] vs. 96.5 [15.8], *p* = 0.110, Student *t* test), and an equal result was found for chronic colonization (FEV1% mean 88.4 [21.4] vs., 98.3 [17.8], *p* = 0.267, Student *t* test).

Twelve patients reported one or more exacerbations during follow-up (70.6%), and 7 of them were frequent exacerbators (41.2%). From the 21 exacerbations recorded, S cultures positive were positive for *S. aureus* in 10 episodes (47.6%), and for other PPMs in 6 (35.3%). In 5 cases (23.8%) no PPM was identified in the exacerbation. Neither the appearance nor the number of exacerbations were significantly related to positive cultures for *S. aureus* at baseline (*p* = 0.6, Fisher exact test and *p* = 0.531, Mann–Whitney U test, respectively), or to chronic colonization by this PPM (*p* = 0.28, Fisher exact test and *p* = 0.095, Mann–Whitney U test, respectively).

### Sequencing

One hundred and seven samples were suitable for sequencing, 48 of them obtained at baseline (16 S, 17 OP and 15 N) and 59 during follow-up (45 S and 14 OP). After removal of low-quality reads and procedure contaminants, a median of 3500 (IQR: 495–13386) reads were recovered from the processed samples, which allowed the identification of a median of 43 genera per sample (IQR: 33–57).

At phylum level, OTUs with higher median RA in S were Firmicutes (36.2%), Bacteroidetes (19.5%), Actinobacteria (11.4%), Fusobacteria (10.3%) and Proteobacteria (7.5%). Gemellaceae genus (10.7%), *Rothia* (8.7%), *Veillonella* (7.5%), *Prevotella* (6.9%) and *Fusobacterium* (5.9%) were the genera with the highest figures in the sample (Supplementary Table S1). These genera were also dominant in one or more of the S samples, together with *Haemophilus* and *Porphyromonas*.

### Topographical Analyses

**FIGURE 1:**
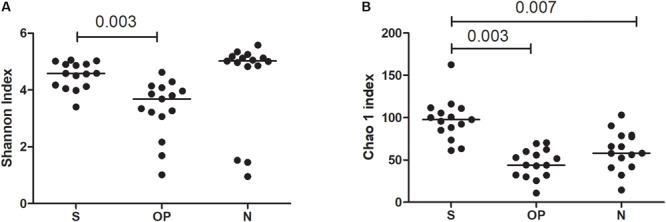
Alpha diversity metrics across sample types. **(A)** Shannon index, **(B)** Chao1 richness estimator. S, sputum; OP, oropharyngeal swab; N, nasal lavage.

**FIGURE 2:**
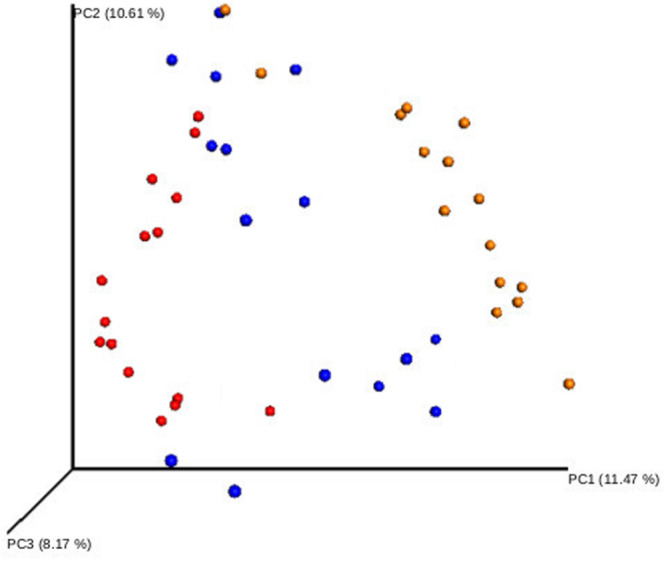
Beta-diversity analysis of the bacterial communities of different respiratory compartments. Principal coordinate analysis (PCoA) of sputum (yellow), oropharyngeal swab (blue), and nasal lavage samples (red). Adonis test of Bray Curtis dissimilarity matrix, *p* = 0.001.

A comparative analysis of the three sampled compartments was performed in the 15 patients with good-quality sequencing results available from the S, N and OP samples obtained at baseline. The diversity of bacterial communities between samples was significantly higher in S than in OP, when measured either with the Shannon index (median 4.6 [IQR: 4.1–4.9] vs. 3.7 [IQR: 3-1-4.1], *p* = 0.003) or the Chao 1 richness estimator (median 97.75 [IQR: 85.1–110.9] vs. 43.9 [IQR: 31.7–59.9], *p* = 0.003). Similar results were obtained when comparing S with N, for the Chao 1 richness estimator (median 97.7 [IQR: 85.1–110.9] vs. 57.9 [41.7–78.6], *p* = 0.005) (Figure 1). Beta-diversity analysis also showed significant differences in the microbial composition of the three bacterial communities (Figure 2) (Adonis test of Bray Curtis dissimilarity matrix, *p* = 0.001).

The predominant phyla in S samples were Firmicutes (32.4%), Bacteroidetes (22.9%), Actinobacteria (14.5%), Fusobacteria (10.0%) and Proteobacteria (8.32%). The most abundant genera in the bronchial compartment were *Prevotella* (12.9%), *Rothia* (11.7%), Gemellaceae genus (11.3%), *Veillonella* (8.8%), *Fusobacterium* (6.8%) and *Porphyromonas* (5.85), which showed higher RAs in S than in samples from other respiratory compartments. This difference reached statistical significance for most of these genera (Table 2).

**TABLE 2:** Genera detected in bronchial tree (S), oropharynx (OP) and nose (N) (median [IQR]).

		**Friedman test**				**Wilcoxon test**	
**Phylum**	**Genus**	**Sputum (S)**	**Oropharyngeal swab (OP)**	**Nasal lavage (N)**	**p**	**S vs. OP**	**S vs. N**
Acidobacteria	Ellin6075_g	0 (0–0)	0 (0–0)	0.21 (0–2.65)	0.002	0.32	0.03
Actinobacteria	*Actinomyces*	1.31 (0.43–1.91)	2.23 (0.06–3.3)	4.17 (0.21–6.38)	0.263	0.12	0.09
Actinobacteria	*Rothia*	11.71 (5.85–23.34)	2.11 (0.8–12.35)	0.53 (0–1.99)	0.001	0.31	0.03
Actinobacteria	0319-7L14_f_g	0 (0–0)	0 (0–0)	1.05 (0–2.46)	0.001	1	0.03
Actinobacteria	*Corynebacterium*	0.04 (0.01–0.11)	0.12 (0–0.64)	0.58 (0–1.57)	0.382	0.25	0.06
Actinobacteria	Nocardioidaceae_g	0 (0–0)	0 (0–0.2)	0.28 (0–1.32)	0.002	0.13	0.03
Actinobacteria	*Propionibacterium*	0 (0–0)	0 (0–0.2)	0.93 (0.17–1.99)	0.002	0.09	0.03
Actinobacteria	*Atopobium*	0.34 (0.05–0.61)	0.23 (0–0.64)	0.93 (0–4.26)	0.789	0.98	0.29
Bacteroidetes	*[Prevotella]*	2.2 (1.72–4.29)	1.66 (0.92–3.51)	0 (0–0)	0.002	0.98	0.03
Bacteroidetes	*Porphyromonas*	5.79 (1.19–7.77)	0.7 (0.06–1.78)	0 (0–2.26)	0.002	0.03	0.03
Bacteroidetes	*Prevotella*	12.88 (5.96–16.13)	8.28 (2.49–14.76)	6.38 (0.26–14.35)	0.024	0.07	0.16
Bacteroidetes	*Capnocytophaga*	1.52 (0.68–2.78)	0.15 (0–0.67)	0 (0–0.01)	0.004	0.03	0.04
Chloroflexi	Gitt-GS-136_o_f_g	0 (0–0)	0 (0–0)	0.53 (0–3.13)	0.002	1	0.03
Firmicutes	Gemellaceae_g	11.32 (6.77–14.34)	2.56 (0.5–3.84)	0.01 (0–1.39)	0.001	0.08	0.03
Firmicutes	*Granulicatella*	4.14 (2.46–5.81)	0.81 (0.32–2.48)	0 (0–1.57)	0.001	0.08	0.03
Firmicutes	*Streptococcus*	0.78 (0.47–1.38)	2.3 (1.37–4.43)	1.06 (0.02–3.83)	0.021	0.03	0.19
Firmicutes	Clostridiales_f_g	0.14 (0.02–0.25)	1.88 (0–3.18)	0 (0–0.35)	0.02	0.03	0.88
Firmicutes	*Veillonella*	8.81 (3.73–12.3)	5.72 (3.76–7.71)	6.53 (0.03–11.5)	0.296	0.09	0.36
Firmicutes	*Staphylococcus*	0.11 (0–0.66)	0 (0–0.03)	0.21 (0–1.58)	0.046	0.04	0.65
Firmicutes	[Mogibacteriaceae]_g	0.25 (0–0.58)	0.82 (0–1.99)	0 (0–1.06)	0.144	0.05	0.96
Firmicutes	*Parvimonas*	0.15 (0–0.17)	0 (0–0.01)	0 (0–0)	0.002	0.03	0.03
Firmicutes	Lachnospiraceae_g	0.68 (0.32–1.07)	0.7 (0.12–1.94)	0.01 (0–3.28)	0.391	0.67	1
Firmicutes	*Catonella*	0.36 (0.03–0.64)	0.1 (0–0.23)	0 (0–0)	0.002	0.09	0.03
Firmicutes	*Moryella*	0.26 (0.03–0.76)	0.2 (0–0.35)	0 (0–0)	0.034	0.32	0.38
Firmicutes	*Oribacterium*	0.86 (0.25–2.54)	0.35 (0–0.75)	0 (0–2.24)	0.077	0.04	0.22
Firmicutes	*Peptostreptococcus*	0.29 (0.1–0.55)	0 (0–0.35)	0 (0–0)	0.002	0.2	0.18
Firmicutes	*Megasphaera*	0.25 (0–1)	0.44 (0–3.31)	0 (0–0)	0.015	0.09	0.12
Firmicutes	*Selenomonas*	0.14 (0.05–0.3)	0.4 (0.1–1.33)	0 (0–0.85)	0.014	0.07	1
Firmicutes	*Bulleidia*	0.22 (0.09–0.71)	0.49 (0.1–2.71)	0 (0–0.7)	0.118	0.2	0.41
Firmicutes	Aerococcaceae_other	0.12 (0–0.43)	0 (0–0)	0 (0–0)	0.012	0.05	0.09
Fusobacteria	*Fusobacterium*	6.81 (2.82–9.76)	2.35 (1.29–7.44)	0 (0–1.28)	0.001	0.25	0.03
Fusobacteria	*Leptotrichia*	3.61 (2.05–8.6)	0.99 (0.15–1.99)	0 (0–1.05)	0.001	0.03	0.03
Planctomycetes	WD2101_f_g	0 (0–0)	0 (0–0)	0.87 (0–1.99)	0.002	1	0.03
Proteobacteria	*Campylobacter*	1.29 (0.35–2.12)	1.38 (0–3.97)	0 (0–0)	0.002	0.46	0.03
Proteobacteria	*Haemophilus*	1.73 (0.58–4.19)	0 (0–0.26)	0 (0–0)	0.001	0.03	0.03
Proteobacteria	*Lautropia*	0.18 (0–0.87)	0 (0–0.03)	0 (0–0)	0.004	0.03	0.04
Proteobacteria	Pseudomonadaceae_g	0 (0–0)	0 (0–0)	0.18 (0–1.99)	0.002	1	0.03
Spirochaetes	*Treponema*	0.11 (0–0.37)	0 (0–0.12)	0 (0–0)	0.015	0.03	0.62
TM7	TM7-1_o_f_g	0 (0–0.05)	0 (0–0.12)	1.05 (0.01–4.63)	0.024	0.42	0.03
TM7	TM7-3_o_f_g	0.59 (0.07–1.25)	4.49 (0.89–30.09)	0.11 (0–1.48)	0.016	0.03	0.86

*Taxa with relative abundances median > 0.1% in at least one type of sample included.*

These results suggest that in early stage CF patients there is higher diversity in the microbiome of the bronchial tree than in the microbial flora of other respiratory compartments, with a clear overrepresentation of specific anaerobe genera in bronchial secretions.

### Longitudinal Study

**FIGURE 3:**
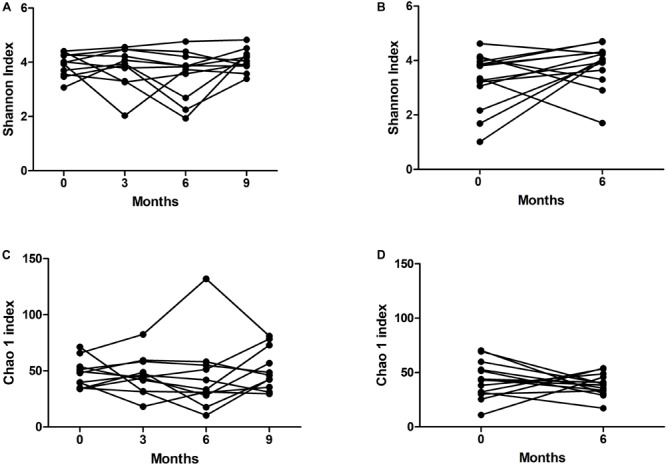
Variability of alpha-diversity over time in **(A,C)** sputum and **(B,D)** oropharyngeal swab samples.

To determine the variability over time in the microbiome of the bronchial tree and oropharynx, we used data from paired S samples from the 17 patients studied, and paired OP samples from 14 patients suitable for sequencing. Alpha-diversity variation in the bacterial flora in S and OP was not statistically significant, using either the Shannon index or the Chao1 richness estimator (Figure 3).

**FIGURE 4:**
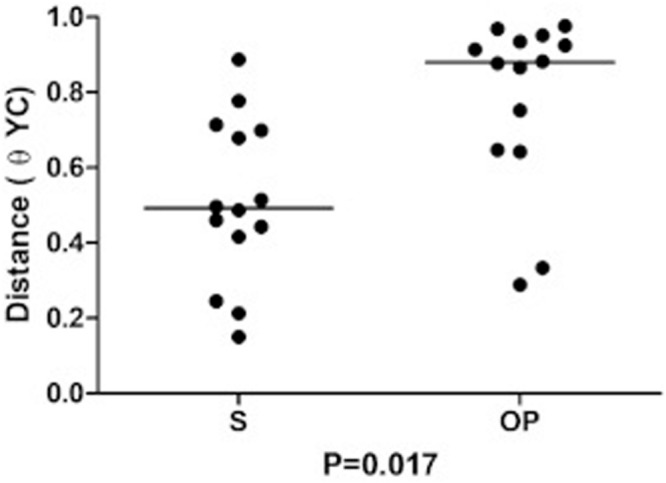
Intrasubject similarity (θ_*YC*_ distance) between bacterial communities recovered at baseline and after 6 months from sputum (S) and from the oropharyngeal swab (OP).

Dissimilarity (θ_*YC*_ distance) of bacterial communities was lower in S than in OP samples obtained after a 6-month interval (*p* = 0.017, Wilcoxon test) (Figure 4). This finding confirms the relative stability of the bacterial flora in the bronchial tree compartment, which was paralleled by the higher temporal variability of oropharyngeal flora, in these patients with early stage CF.

### Bronchial Microbiome and Clinical Patterns

*Staphylococcus aureus* was the main PPM recovered from S cultures at baseline and during the study period, in spite that *Staphylococcus* genus did not reach a dominance pattern in any S sample and showed low RAs in most of them (median 0.26% [IQR 0.01–0.69%]).

**FIGURE 5:**
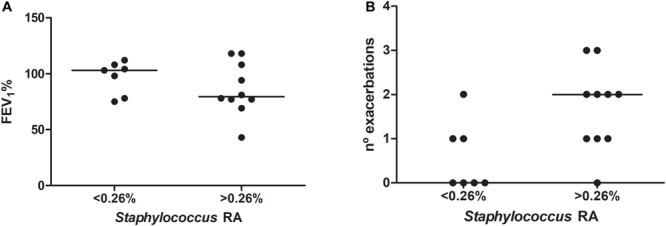
Relationship between the overrepresentation of *Staphylococcus* in bronchial secretions (relative abundance above 0.26% in sputum) and clinical measures: **(A)** forced expiratory volume in the first second as a percentage of the reference (FEV1%); and **(B)** exacerbation frequency during the study period.

A dominance pattern of the genera *Prevotella*, *Veillonella*, *Gemellaceae* genus, *Porphyromonas*, *Rothia* or *Haemophilus* was observed in five patients at baseline and was significantly associated with FEV1 impairment (FEV1%, mean 74.8 [SD 19] vs. 97.2 [SD 17.8], *p* = 0.035, Student *t* test). Patients with higher *Staphylococcus* median RAs (>0.26%) at baseline showed lower FEV1, but this difference did not reach statistical significance (FEV1% mean 86.3 [SD 23.5] vs. 96.9 [14.6], *p* = 0.311, Student *t* test) (Figure 5). Testing the other taxa with median RAs above 0.1% in S at baseline (*n* = 31) gave similar non-significant results (data not shown).

Patients with *Staphylococcus* RAs over 0.26% at baseline suffered more exacerbations (median 2 [IQR 1–2.25] vs. 0 [0–1]) (Figure 5), often due to *S. aureus* (10/17, 58.8%), and higher RAs were also significantly associated chronic colonization by *S. aureus* (9/10 [90%] vs. 2/7 [28.6%], *p* = 0.034, Fisher’s exact test). This trend was not observed when the relationship with exacerbation frequency was tested for the other taxa (Supplementary Table S2). Dominance patterns at baseline were not associated with higher exacerbation frequencies during follow-up (median 1 [IQR 1–2] vs. 1 [0–2], *p* = 0.701, Mann–Whitney U test).

These observations suggest that *Staphylococcus* genus RAs above 0.26%, in spite of being low and far from dominance patterns, are clinically meaningful, because they are associated with the subsequent appearance *S. aureus-*related acute episodes and the persistence of this PPM as colonizer, together with a tendency to suffer exacerbations more frequently.

## Discussion

In the present cohort study, the microbiome of the bronchial tree and two additional upper respiratory compartments, the nose and the oropharynx, was assessed in early stage stable CF patients not using chronic antibiotic therapy. *S. aureus* was recovered from S cultures during the study in more than a half of the participants, but all samples were negative for *P. aeruginosa* and other common CF colonizers. The bronchial microbiome showed a significantly higher diversity than oropharynx and nose. In spite that the RA of the *Staphylococcus* genus was low in bronchial secretions and did not reach 1% in most patients, participants with RAs above 0.26% suffered more exacerbations, in most cases due to *S. aureus*, that often persists as chronic colonizer in the bronchial tree, confirming the clinical meaningfulness of small overrepresentations of the *Staphylococcus* genus, even at low RAs.

In the present study Firmicutes, Bacteroidetes, Actinobacteria, and Fusobacteria were the predominant phylum in bronchial secretions, with *Prevotella, Rothia* and Gemellaceae genus as the most abundant genera. The genera *Pseudomonas* and *Burkholderia* were not identified in the studied respiratory samples, in spite that *P. aeruginosa* and *Burkholderia* species are commonly cultured from CF patients (Guss et al., 2011; Fodor et al., 2012). Common PPMs are typically acquired in temporal succession in CF, beginning early in life with *S. aureus* and then progressing to recurrent infections dominated by *P. aeruginosa* and *B. cepacia* complex species (Razvi et al., 2009; Huang and Lynch, 2011), which often persist as colonizers and reach a pattern of dominance with RAs over 50% (Fodor et al., 2012). The frequent recovery of *S. aureus* from bronchial secretions in the present study in the absence of *P. aeruginosa* is expected considering that enrolled patients showed early stage disease and did not use chronic antibiotic treatments.

The bronchial microbiome had higher diversity than the microbial flora in oropharynx and nose in the present study. These results emphasize the need to obtain bronchial samples for an accurate evaluation of the bronchial flora in early stage patients, to avoid any misevaluation that may derive from the single assessment of oropharyngeal microbiome. In CF the bacterial composition of the oropharynx has been often considered representative of the bronchial flora as in healthy subjects (Fodor et al., 2012; Boutin et al., 2015; Dickson et al., 2015). However, previous studies on CF patients have included a high proportion of patients at intermediate or advanced stages of the disease (FEV1% < 70%) often colonized by *P. aeruginosa* (Fodor et al., 2012; Boutin et al., 2015), a pattern mainly observed in adult patients (Coburn et al., 2015). The results of the present study suggests that the microbial composition of bronchi and oropharynx have significant differences when the disease is at an early stage in younger patients, through an overrepresentation of specific genera in bronchial secretions, in accordance with previous suggestions (Zemanick et al., 2015, 2017).

A dominant pattern was observed at baseline in near a third of the participants and was related to lung function impairment. Dominance was mainly due to increased RAs of different anaerobic genera as are *Prevotella*, *Veillonella*, *Gemella*, *Porphyromonas* or *Rothia*, in the present study, and less often related to the overrepresentation of aerobic genus as *Haemophilus*. These results confirm the clinical meaning of dominance patterns, even when unrelated to *Pseudomonas*, *Burkholderia*, *Stenotrophomonas*, and *Achromobacter*, the genera most commonly showing high RAs, which when dominant are often associated with diversity declines, low lung volumes and chronic colonization (Delhaes et al., 2012; Coburn et al., 2015; Flight et al., 2015). Dominance patterns were not related to exacerbation frequency in the present study, in agreement with previous studies that have mainly related the appearance of exacerbations to changes in the microbial composition of bronchial secretions severe enough to be considered as disbiosis, while dominant taxa are mainly observed is stable condition when the disease is advanced (Carmody et al., 2013, 2015).

*Staphylococcus aureus* was recovered from one third of the S cultures during the study period, even though the *Staphylococcus* genus had RAs mostly below 1% and did not attain a dominant pattern. Patients with RAs over 0.26% for this genus did not shown a lung function impairment, but suffered more exacerbations during follow-up, due to *S. aureus* in more than a half of the cases, which often persist as chronic colonization. This finding suggests that small overrepresentations of the *Staphylococcus* genus may have clinical significance, even when showing low RAs. Additional studies would be needed to confirm these results.

The present study has included 17 patients, that may not be representative of the CF population as a whole, as other studies of the respiratory microbiome of CF with a similar number of participants (Fodor et al., 2012; Boutin et al., 2015). It is important also to consider that the study is restricted to early stage CF, and does not include CF patients with intermediate and advanced disease, and the results obtained cannot be extrapolated to this population. In most cases these patients require chronic and/or recurrent antibiotherapy, a treatment whose impact on the respiratory microbiome has been demonstrated (Carmody et al., 2018). The acceptance of this limiting selection criterion has the advantage of allowing a detailed study of respiratory flora at earlier stages of the disease and outside the use of regular antibiotics, a period that has been only marginally studied.

To conclude, the bronchial microbiome shows higher diversity than the microbial flora lodged in the oropharynx and nose in early stage CF, mainly through an overrepresentation of anaerobic genera in bronchial secretions. Different taxa may begin to show dominance patterns in this clinical stage, and, in spite of their variability, were associated with identifiable functional impairments. *S. aureus* was often cultured from these early stage CF patients, but the *Staphylococcus* genus shows only low RAs in bronchial secretions. However, patients with slight overrepresentations of this genus, even at rates below 1%, suffered more exacerbations, mostly due to *S. aureus*, and frequently evolve to a chronic colonization pattern by this PPM, findings that confirm the importance of the appearance of that genus in bronchial secretions, regardless of its abundance.

## Data Availability Statement

The datasets generated for this study can be found in the Bacterial 16S rRNA, data sets from this study are accessible in the European Nucleotide Archive under the reference PRJEB31332 with the sample numbers ERS3193233–ERS3193339 (http://www.ebi.ac.uk/ena/data/view/ERS3193233-ERS3193339).

## Ethics Statement

All clinical investigations were conducted in accordance with the principles expressed in the Declaration of Helsinki. The studies involving human participants were reviewed and approved by The Parc Taul University Hospital Ethics Board. Written informed consent to participate in this study was provided by participants or by the participants’ legal guardian/next of kin. Furthermore, protocols have been adhered to standard biosecurity and institutional safety procedures in this study.

## Author Contributions

MG-N, MB, XP, and EM conceived and designed the study. XP, CM, and MB contributed to the recruitment of the participants. XP, CM, MG-G, and EP acquisition of the clinical data. MG-N, LM, SQ, SC, and OC contributed to the reagents, materials, and analysis tools. MG-N and EM did the statistical analysis, prepared the figures and tables and wrote the first draft which was reviewed and approved by all the co-authors. OA, MB, and MG-G critically reviewed and revised the manuscript for important intellectual content. All authors contributed to the interpretation of the results and approved the final version for publication.

## Conflict of Interest

The authors declare that the research was conducted in the absence of any commercial or financial relationships that could be construed as a potential conflict of interest.
