# Supplementary material for: The Respiratory Microbiome in Cystic Fibrosis: Compartment Patterns and Clinical Relationships in Early Stage Disease
Source: Front Microbiol. 2020 Jun 30;11:1463. doi: 10.3389/fmicb.2020.01463 (PMC7339930; doi:10.3389/fmicb.2020.01463)
Supplement: Supplementary file 1 [file Table_1.docx]

**The respiratory microbiome in cystic fibrosis: compartment patterns and clinical relationships in early-stage disease**

Marian Garcia-Nuñez^1,2^, Miguel Garcia-Gonzalez^3,4,5^, Xavier Pomares^1,2,3^, Concepción Montón^1,3^, Laura Millares^1,2,6^, Sara Quero^1,2,6^, Elena Prina^1^, Oscar Asensio^3,4^, Montserrat Bosque^3,4^, Silvia Capilla^7^, Oscar Cuevas^4^, Eduard Monsó^1,2,5,*^.

Table S1. Genera detected in bronchial tree (S), oropharynx (OP) and nose (N) (median [IQR]). Taxa with ≥30% of occurrence in at least one type of sample included. Results expressed as median (interquartile range).

| **Phylum** | **Genus** | **S** | **OP** | **N** |
| --- | --- | --- | --- | --- |
| Acidobacteria | Ellin6075_g | 0 (0-0) | 0 (0-0) | 0.21 (0-2.65) |
| Acidobacteria | Solibacterales_f_g | 0 (0-0) | 0 (0-0) | 0 (0-0.37) |
| Actinobacteria | *Actinomyces* | 1.31 (0.43-1.91) | 2.23 (0.06-3.3) | 4.17 (0.21-6.38) |
| Actinobacteria | *Rothia* | 11.71 (5.85-23.34) | 2.11 (0.8-12.35) | 0.53 (0-1.99) |
| Actinobacteria | 0319-7L14_f_g | 0 (0-0) | 0 (0-0) | 1.05 (0-2.46) |
| Actinobacteria | *Corynebacterium* | 0.04 (0.01-0.11) | 0.12 (0-0.64) | 0.58 (0-1.57) |
| Actinobacteria | Nocardioidaceae_g | 0 (0-0) | 0 (0-0.2) | 0.28 (0-1.32) |
| Actinobacteria | *Propionibacterium* | 0 (0-0) | 0 (0-0.2) | 0.93 (0.17-1.99) |
| Actinobacteria | *Atopobium* | 0.34 (0.05-0.61) | 0.23 (0-0.64) | 0.93 (0-4.26) |
| Actinobacteria | *Kocuria* | 0 (0-0) | 0 (0-0) | 0 (0-0.04) |
| Actinobacteria | Propionibacteriaceae_g | 0 (0-0.01) | 0 (0-0) | 0 (0-0) |
| Actinobacteria | Pseudonocardiaceae_g | 0 (0-0) | 0 (0-0) | 0 (0-0.26) |
| Actinobacteria | *Pseudonocardia* | 0 (0-0) | 0 (0-0) | 0 (0-1.32) |
| Actinobacteria | Gaiellaceae_g | 0 (0-0) | 0 (0-0.12) | 0 (0-0.09) |
| Bacteroidetes | [Prevotella] | 2.2 (1.72-4.29) | 1.66 (0.92-3.51) | 0 (0-0) |
| Bacteroidetes | *Porphyromonas* | 5.79 (1.19-7.77) | 0.7 (0.06-1.78) | 0 (0-2.26) |
| Bacteroidetes | *Prevotella* | 12.88 (5.96-16.13) | 8.28 (2.49-14.76) | 6.38 (0.26-14.35) |
| Bacteroidetes | *Capnocytophaga* | 1.52 (0.68-2.78) | 0.15 (0-0.67) | 0 (0-0.01) |
| Bacteroidetes | *Tannerella* | 0.1 (0-0.24) | 0 (0-0.24) | 0 (0-0) |
| Bacteroidetes | Chitinophagaceae_g | 0 (0-0) | 0 (0-0.2) | 0 (0-1.42) |
| Bacteroidetes | *Adhaeribacter* | 0 (0-0) | 0 (0-0) | 0 (0-0.35) |
| Bacteroidetes | [Weeksellaceae]_g | 0.02 (0-0.11) | 0 (0-0) | 0 (0-0) |
| Chloroflexi | AKIW781_f_g | 0 (0-0) | 0 (0-0.23) | 0 (0-0.17) |
| Chloroflexi | Gitt-GS-136_o_f_g | 0 (0-0) | 0 (0-0) | 0.53 (0-3.13) |
| Cyanobacteria | Streptophyta_f_g | 0 (0-0.01) | 0.07 (0-0.99) | 0.04 (0-1.99) |
| Firmicutes | Gemellaceae_g | 11.32 (6.77-14.34) | 2.56 (0.5-3.84) | 0.01 (0-1.39) |
| Firmicutes | *Granulicatella* | 4.14 (2.46-5.81) | 0.81 (0.32-2.48) | 0 (0-1.57) |
| Firmicutes | *Streptococcus* | 0.78 (0.47-1.38) | 2.3 (1.37-4.43) | 1.06 (0.02-3.83) |
| Firmicutes | Clostridiales_f_g | 0.14 (0.02-0.25) | 1.88 (0-3.18) | 0 (0-0.35) |
| Firmicutes | *Veillonella* | 8.81 (3.73-12.3) | 5.72 (3.76-7.71) | 6.53 (0.03-11.5) |
| Firmicutes | *Staphylococcus* | 0.11 (0-0.66) | 0 (0-0.03) | 0.21 (0-1.58) |
| Firmicutes | *Gemella* | 0.08 (0-0.14) | 0 (0-0.13) | 0 (0-0) |
| Firmicutes | [Mogibacteriaceae]_g | 0.25 (0-0.58) | 0.82 (0-1.99) | 0 (0-1.06) |
| Firmicutes | *Mogibacterium* | 0.05 (0.02-0.12) | 0 (0-0.05) | 0 (0-0) |
| Firmicutes | *Parvimonas* | 0.15 (0-0.17) | 0 (0-0.01) | 0 (0-0) |
| Firmicutes | Lachnospiraceae_g | 0.68 (0.32-1.07) | 0.7 (0.12-1.94) | 0.01 (0-3.28) |
| Firmicutes | *Butyrivibrio* | 0 (0-0.15) | 0 (0-0.12) | 0 (0-0) |
| Firmicutes | *Catonella* | 0.36 (0.03-0.64) | 0.1 (0-0.23) | 0 (0-0) |
| Firmicutes | *Moryella* | 0.26 (0.03-0.76) | 0.2 (0-0.35) | 0 (0-0) |
| Firmicutes | *Oribacterium* | 0.86 (0.25-2.54) | 0.35 (0-0.75) | 0 (0-2.24) |
| Firmicutes | *Peptostreptococcus* | 0.29 (0.1-0.55) | 0 (0-0.35) | 0 (0-0) |
| Firmicutes | *Megasphaera* | 0.25 (0-1) | 0.44 (0-3.31) | 0 (0-0) |
| Firmicutes | *Selenomonas* | 0.14 (0.05-0.3) | 0.4 (0.1-1.33) | 0 (0-0.85) |
| Firmicutes | *Bulleidia* | 0.22 (0.09-0.71) | 0.49 (0.1-2.71) | 0 (0-0.7) |
| Firmicutes | Planococcaceae_g | 0.02 (0-0.09) | 0 (0-0) | 0 (0-0) |
| Firmicutes | *Lysinibacillus* | 0 (0-0) | 0 (0-0.15) | 0 (0-0) |
| Firmicutes | Gemellales_f_g | 0.01 (0-0.07) | 0 (0-0) | 0 (0-0) |
| Firmicutes | Gemellaceae_other | 0 (0-0.01) | 0 (0-0) | 0 (0-0) |
| Firmicutes | Aerococcaceae_other | 0.12 (0-0.43) | 0 (0-0) | 0 (0-0) |
| Firmicutes | *Vagococcus* | 0.01 (0-0.02) | 0 (0-0) | 0 (0-0) |
| Firmicutes | Streptococcaceae_g | 0.01 (0-0.03) | 0 (0-0) | 0 (0-0) |
| Firmicutes | Lactobacillales_Other_other | 0 (0-0.06) | 0 (0-0) | 0 (0-0) |
| Firmicutes | Bacilli_other_other_other | 0 (0-0.01) | 0 (0-0) | 0 (0-0) |
| Firmicutes | *Peptococcus* | 0.03 (0-0.05) | 0 (0-0) | 0 (0-0) |
| Firmicutes | Peptostreptococcaceae_g | 0.01 (0-0.02) | 0 (0-0) | 0 (0-0) |
| Firmicutes | *Filifactor* | 0 (0-0.23) | 0 (0-0) | 0 (0-0) |
| Firmicutes | *Dialister* | 0 (0-0.06) | 0 (0-0) | 0 (0-0) |
| Fusobacteria | *Fusobacterium* | 6.81 (2.82-9.76) | 2.35 (1.29-7.44) | 0 (0-1.28) |
| Fusobacteria | *Leptotrichia* | 3.61 (2.05-8.6) | 0.99 (0.15-1.99) | 0 (0-1.05) |
| Fusobacteria | Leptotrichiaceae_g | 0.03 (0-0.41) | 0 (0-0.41) | 0 (0-0) |
| Planctomycetes | WD2101_f_g | 0 (0-0) | 0 (0-0) | 0.87 (0-1.99) |
| Proteobacteria | *Campylobacter* | 1.29 (0.35-2.12) | 1.38 (0-3.97) | 0 (0-0) |
| Proteobacteria | *Haemophilus* | 1.73 (0.58-4.19) | 0 (0-0.26) | 0 (0-0) |
| Proteobacteria | *Lautropia* | 0.18 (0-0.87) | 0 (0-0.03) | 0 (0-0) |
| Proteobacteria | Comamonadaceae_g | 0 (0-0) | 0 (0-0.24) | 0.09 (0-1.06) |
| Proteobacteria | *Agrobacterium* | 0 (0-0) | 0 (0-0) | 0.1 (0-0.93) |
| Proteobacteria | Rhodospirillaceae_g | 0 (0-0) | 0 (0-0.16) | 0 (0-0) |
| Proteobacteria | Neisseriales_f_g | 0.01 (0-0.57) | 0 (0-0) | 0 (0-0) |
| Proteobacteria | *Eikenella* | 0.02 (0-0.29) | 0 (0-0) | 0 (0-0) |
| Proteobacteria | *Neisseria* | 0.01 (0-0.04) | 0 (0-0) | 0 (0-0) |
| Proteobacteria | *Cardiobacterium* | 0.03 (0-0.08) | 0 (0-0) | 0 (0-0) |
| Proteobacteria | *Actinobacillus* | 0.09 (0-5.09) | 0 (0-0) | 0 (0-0) |
| Proteobacteria | *Aggregatibacter* | 0 (0-0.09) | 0 (0-0) | 0 (0-0) |
| Proteobacteria | Pseudomonadaceae_g | 0 (0-0) | 0 (0-0) | 0.18 (0-1.99) |
| Proteobacteria | PseudomoN | 0 (0-0) | 0 (0-0) | 0 (0-1.04) |
| Spirochaetes | *Treponema* | 0.11 (0-0.37) | 0 (0-0.12) | 0 (0-0) |
| SR1 | SR1_c_o_f_g | 0 (0-0.07) | 0 (0-0) | 0 (0-0) |
| Tenericutes | *Mycoplasma* | 0.01 (0-0.1) | 0 (0-0) | 0 (0-0) |
| TM7 | TM7-1_o_f_g | 0 (0-0.05) | 0 (0-0.12) | 1.05 (0.01-4.63) |
| TM7 | TM7-3_o_f_g | 0.59 (0.07-1.25) | 4.49 (0.89-30.09) | 0.11 (0-1.48) |
| TM7 | F16_g | 0.09 (0.01-0.96) | 0 (0-0.4) | 0 (0-1.42) |
| TM7 | CW040_f_g | 0 (0-0.14) | 0 (0-0) | 0 (0-0) |
