# Supplementary material for: The Respiratory Microbiome in Cystic Fibrosis: Compartment Patterns and Clinical Relationships in Early Stage Disease
Source: Front Microbiol. 2020 Jun 30;11:1463. doi: 10.3389/fmicb.2020.01463 (PMC7339930; doi:10.3389/fmicb.2020.01463)
Supplement: Supplementary file 2 [file Table_2.DOCX]

Table S2.- Significance of the association of taxa with relative abundance over 0.1% in sputum at baseline and exacerbation frequency the following 9 months.

|  | **Mann-Whitney U test**  **p** | **Benjamini-Hochberg Adjusted P value** |
| --- | --- | --- |
| ***Staphylococcus*** | **0.026** | **0.832** |
| ***Peptostreptococcaceae_g*** | 0.085 | 1 |
| ***Capnocytophaga*** | 0.121 | 0.968 |
| ***Fusobacterium*** | 0.121 | 1 |
| ***Aerococcaceae_g*** | 0.193 | 1 |
| ***Clostridiales*** | 0.230 | 1 |
| ***Tannerella*** | 0.294 | 1 |
| ***Lachnospiraceae*** | 0.294 | 1 |
| ***Leptotrichia*** | 0.294 | 1 |
| ***Haemophilus*** | 0.342 | 1 |
| ***Moryella*** | 0.388 | 1 |
| ***Oribacterium*** | 0.424 | 1 |
| ***Parvimonas*** | 0.424 | 1 |
| ***Treponema*** | 0.484 | 1 |
| ***Rothia*** | 0.510 | 1 |
| ***[Mogibacteriaceae]*** | 0.541 | 1 |
| ***Granulicatella*** | 0.582 | 1 |
| ***Atopobium*** | 0653 | 1 |
| ***Lautropia*** | 0.653 | 1 |
| ***TM7-3_g*** | 0.653 | 1 |
| ***Megasphaera*** | 0.723 | 1 |
| ***Selenomonas*** | 0.723 | 0.964 |
| ***Veillonella*** | 0.723 | 1 |
| ***Campylobacter*** | 0.723 | 0.925 |
| ***Streptococcus*** | 0.723 | 1 |
| ***Gemellaceae_g*** | 0.761 | 0.937 |
| ***Porphyromonas*** | 0.764 | 0.905 |
| ***Bulleidia*** | 0.764 | 0.873 |
| ***Catonella*** | 0.841 | 0.930 |
| ***Actinomyces*** | 0.879 | 0.938 |
| ***Prevotella*** | 0.920 | 0.950 |
| **[Prevotella]** | 0.960 | 0.96 |
